# Work-related olfactory disorder: a case series and review

**DOI:** 10.1186/s40557-018-0230-3

**Published:** 2018-03-12

**Authors:** Soon Woo Park, Young Joong Kang, Huisu Eom, Hyun-Jin Cho, Jungho Ahn, Sang-Gil Lee

**Affiliations:** 1 Occupational Safety and Health Research Institute, Korea Occupational Safety and Health Agency, 400, Jongga-ro, Jung-gu, Ulsan, 44429 South Korea; 2 Department of Otorhinolaryngology, Gyeongsang National University Hospital, Jinju, South Korea; 3 Institute of Health Sciences, Gyeongsang National University, Jinju, South Korea

**Keywords:** Olfactory disorder, Occupational disease, Anosmia, Work-relatedness

## Abstract

**Background:**

The olfactory bulb is anatomically exposed and thus can be directly damaged by external stimulation. This can occur as an occupational injury owing to contact with organic solvents or other causes. We present cases of eight patients who sustained occupation-related exposure to potentially toxic substances and later presented with signs and symptoms of anosmia. We examined the occupational and medical characteristics of the patients and evaluated their work-relatedness.

**Case presentation:**

Case 1: A 50-year-old man performed high-frequency heat treatments for approximately 11 years. He experienced decreased senses for olfaction and taste during the later years culminating in the diagnosis of anosmia after 3 years (high work-relatedness). Case 2: A 54-year-old man whose work involved exposure to various organic solvents, such as spray painting and application of paint and thinners for approximately 4 years, was subsequently diagnosed with anosmia based on rhinorrhea, headache, and loss of olfaction (high work-relatedness). Case 3: A 44-year-old-man who performed spray painting for approximately 17 years developed anosmia (high work-relatedness). Case 4: A 44-year-old man was involved in ship engine cleaning once a month, for approximately 7 h per cleaning session; he was diagnosed with anosmia based on loss of olfaction (low work-relatedness). Case 5: A 41-year-old man worked in ship building block construction for approximately 13 years; anosmia diagnosis was based on loss of olfaction (low work-relatedness). Case 6: A 47-year-old woman performed product inspection and labeling at a plant manufacturing automobile parts; anosmia diagnosis was based on decreased olfaction and taste (low work-relatedness). Case 7: A 50-year-old woman performed epoxy coating in a plant manufacturing automobile parts; anosmia diagnosis was based on diminishing olfaction (low work-relatedness). Case 8: A 57-year-old woman performed cleaning of the area where mobile phone parts were manufactured; anosmia diagnosis was based on diminishing olfaction (low work-relatedness).

**Conclusion:**

The study results confirmed work-relatedness when the subject was young, and the duration of exposure was long without any other cause of anosmia. Regarding compensation for occupational diseases, work-relatedness can be recognized as a relative concept.

## Background

Olfactory disorder has been less of a medical concern compared with visual or auditory disturbances because the symptoms of the disorder can be subtle and less immediately apparent, compared with disorders of the other senses [1]. Nevertheless, the quality of life is severely impaired in patients with chronic severe hyposmia or anosmia [2]. Olfaction plays a significant role in our ability to detect the aroma and flavor of foods. It is also responsible for our awareness of environmental fragrances.

The olfactory sensory neurons are true bipolar neurons that have anatomically different features in contrast to the peripheral components of the auditory and visual systems. The olfactory bulb is directly exposed to the external environment and thus can be vulnerable to external stimulation from inflammatory, infectious, and chemical agents. Under normal conditions, the olfactory sensory neurons undergo regular turnover of receptor cells via apoptotic cell death; however, in case of an olfactory disorder, this balance can be disturbed with an unbalanced loss of olfactory sensory neurons owing to direct stimulation with organic solvents or by other causes [3]. Olfactory disorders are not only important sensory nerve injuries but also an indicator of nerve damage due to external stimulation [4].

Olfactory disorder is regarded as a common and mild disorder, but there is a lack of critical disease information, such as consistent epidemiology statistics, risk factor, and occupational/environmental-relatedness. The pathophysiology of olfactory disorder and causative agents, such as organic solvents and heavy metals, are relatively well known, but critical disease information on dose–response relationship is lacking.

There are few studies on olfactory nerve damage related to occupational exposure in Korea. It is necessary to study olfactory nerve damage caused by occupational exposure and to consider not only workers’ health and disease prevention techniques, but also workers’ compensation in cases of irreversible olfactory nerve damage.

We reviewed reports of eight potentially work-related cases of anosmia (2001–2016), which required investigation by the Occupational Safety and Health Research Institute (OSHRI) in the Korea Occupational Safety and Health Agency (KOSHA). All investigations were based on a request from the Korea Workers Compensation and Welfare Service (KCOMWEL) for the compensation of occupational disease between 2001 and 2016. We examined the occupational and medical characteristics of the eight anosmia cases and evaluated their work-relatedness. We also considered factors that may improve future research on work-related anosmia in our review (Table 1).

**Table 1:** Detailed history and examination of 8 anosmia cases

Case No.	Sex	Age (yr)	Job	Potential occupational exposure	Length of exposure	Olfactory test	Imaging study	Smoking	Work-relatedness*
1	M	50	High Frequency Heat Treatment Workers	Organic solvent	11yrs	Olfactory identification test and olfactory threshold test	PNS series – non-specific	5 pack years	High
2	M	39	Spray painting	Organic solvent	4yrs, 3months	Olfactory identification test and olfactory threshold test	None	15 pack years	High
3	M	44	Spray painting	Organic solvent	17yrs	Olfactory identification test and olfactory threshold test	PNS CT - non-specific Brain MRI - non-specific	None	High
4	M	44	Ship engine cleaning	Organic solvent	21yrs	Olfactory identification test and olfactory threshold test	PNS CT - chronic sinusitis MRI - frontal lobe encephalomalacia	20 pack years	Low
5	M	41	Welder	Welding fumes	13yrs, 2months	Olfactory identification test and olfactory threshold test	PNS CT - chronic sinusitis Brain MRI - non-specific	22 pack years	Low
6	F	47	Spray adhesive labeling job	Volatile organic compounds	11months	Olfactory identification test and olfactory threshold test	PNS CT - non-specific Sinus endoscopy- non-specific	None	Low
7	F	50	Automobile parts epoxy coating	Organic solvent	2yrs, 9months	Olfactory identification test and olfactory threshold test	PNS CT- Mild, Left side nasal septal deviation Sinus endoscopy- non-specific	None	Low
8	F	57	Clean room cleaning	Organic compound (cyclohexanone)	1yr, 2months	Olfactory identification test and olfactory threshold test	None	None	Low

*Occupational Safety and Health Research Institute (OSHRI) investigated the work relatedness for the compensation of high work-related disease

## Case presentation

### Case 1 (M/50): High-frequency heat treatment workers, organic solvents

This worker performed high-frequency heat treatments (induction hardening, a form of heat treatment in which a metal part is heated by induction heating and then quenching) for approximately 11 years (1995–2006), at a factory manufacturing automobile parts. In 2003, he noticed his sense of smell and taste had decreased. In 2006, he visited the otorhinolaryngology department with the above symptoms and was diagnosed with anosmia following an olfactory identification and threshold test. At that time, the paranasal sinus (PNS) radiographs did not show abnormalities, and we observed no inflammation or ulceration in the nasal cavities and sinuses, which could cause olfactory disorders. There was no evidence of a long-term treatment for allergic rhinitis or acute upper respiratory infection upon examination of the employee’s medical insurance check. The worker smoked for 10 consecutive years (5 pack-years) and quit smoking at 5 years before anosmia diagnosis.

Heat treatment (or treating) refers to industrial and metal processes that alter the physical and chemical properties of materials by heating or cooling. High-frequency heat treatments is a type of heat treatment that alter only the necessary area of the material with electromagnetic induction and improve the abrasion resistance of a metal. The process uses chemical treatment agent for cooling and rust preventive oil. Bulk samples of the heat treatment agent and rust preventive oil used by workers included organic solvents such as octamethyl-cyclotetrasiloxane, salicylic acid, benzoic acid, methenamine, methylamine, and butanol.

#### Commentary

As a result of his occupational history, the worker had an 11-year history of exposure to organic solvents that could potentially damage the olfactory nerves. No other cause of anosmia, aside from the organic solvents, could be found. The investigators determined that, in this case, the burden of occupational exposure was sufficient to cause severe anosmia.

### Case 2 (M/54): Spray painting, organic solvents

Between 1996 and 2000, this individual worked in a bicycle manufacturing plant. His work involved exposure to various organic solvents such as spray painting as well as applying paint and thinners. Since August 1999, he complained of rhinorrhea, headache, and loss of smell. These persisted to presentation 3 years and 6 months later. In September 2000, he was diagnosed with anosmia following an olfactory identification and threshold test at the general hospital. There was no past history of otorhinolaryngological disorders. He smoked 15 cigarettes a day for approximately 20 years (15 pack-years); although not a binge drinker, he had been drinking for 20 years.

Through his work, he was exposed to hazardous substances such as n-butyl alcohol, ethyl benzene, indole-3-butyric acid, isopropyl alcohol, methyl ethyl ketone, tetrachloroethylene, 1,1,1-trichloroethane, toluene, ρ-, m-, ο-xylene, and petroleum naphtha. To confirm work-relatedness, investigators evaluated the work environment and found the exposure level to be below the permissible exposure limit. However, this worker voluntarily took on additional work during weekends. Investigators estimated that actual organic solvent exposure levels were higher than investigators’ previous measures because work time, work intensity, and workload were higher than those at the time of the original investigation.

#### Commentary

The results of occupational history and work environment evaluation confirmed that this worker was exposed to composite organic solvents. Investigators have not found any cause aside from organic solvent exposure and the worker’s anosmia appeared to be related to exposure to composite organic solvents during painting.

### Case 3 (M/44): Spray painting, organic solvents

This individual performed spray painting for approximately 17 years, from 1998 to 2014, at a plant manufacturing automobile parts. Three years after he started spray painting, his sense of smell began to diminish and his symptoms gradually worsened. In 2014, he was diagnosed with anosmia as a result of an olfactory identification and threshold test. Although symptoms of allergic rhinitis and sinusitis had been reported since 1999, there were no positive findings for chronic rhinitis or sinusitis on brain MRI and PNS CT, performed in 2015. There was no record of chronic rhinitis or sinusitis in his health insurance records. There was no past medical history and smoking history.

The worker performed spray painting. Organic solvents such as toluene, xylene, ethylbenzene, acetone, and n-hexane were detected in both personal and local exposure measurement samples and were 10 to 30% of the permissible exposure limit. However, at the time of the investigation, automated painting had partially taken over the painting process. In the past, painting was mostly performed manually. Consequently, work time, work intensity, and workload were higher in the past than those at the time of the investigation. Thus, historical exposure levels to organic solvents were likely higher than contemporary measures.

#### Commentary

This worker had a nearly two-decade history of spray painting during which time he was exposed to organic solvents and handled acetone and paint. There were no other underlying diseases or risk factors that could have caused anosmia. Investigators determined that the burden of occupational exposure was sufficient to cause the worker’s anosmia.

### Case 4 (M/44): Ship engine cleaning, organic solvents

This individual worked in ship engine assembly shop for 21 years, from 1984 to 2005, where he carried out ship engine cleaning work once a month, for approximately 7 h per cleaning. In 2004, he visited the general hospital with loss of smell and was diagnosed with anosmia as a result of olfactory identification and threshold tests. There was a history of head trauma and frontal lobe surgery 10 years prior to presentation. Brain MRI and PNS CT were performed in 2006 when the patient was checked for frontal lobe encephalomalacia and chronic sinusitis. He smoked for approximately 20 years (20 pack-years), and had no other significant past medical history, aside from the brain surgery.

The washing fluid Material Safety Data Sheets (MSDS) revealed the solution consisted of organic solvents such as n-butyl alcohol, toluene, xylene, and tri-methylbenzene. Exposure levels of n-butyl alcohol, toluene, xylene, and tri-methylbenzene were all below the permissible exposure limit, in both personal and local samples.

#### Commentary

The worker has been cleaning ship engines for 21 years and was likely exposed to organic solvents. However, the cleaning work was performed only once a month, for approximately 7 h per session, and the organic solvents exposure levels were as low as 1 ppm. Therefore, it was difficult to determine if he was continuously exposed to organic solvents for 21 years. In addition, there was a history of brain surgery due to head trauma 10 years before his anosmia diagnosis. MRI and CT scans after the diagnosis of anosmia revealed frontal lobe encephalomalacia and chronic sinusitis. Through a literature review the investigators confirmed the relationship between chronic sinusitis and frontal lobe encephalomalacia and anosmia. Overall, the investigators judged that the cause of anosmia was likely chronic sinusitis and encephalomalacia. Investigators determined that the burden of occupational exposure was insufficient to cause anosmia.

### Case 5 (M/41): Welder, welding fume

This worker was a steel block welder in ship building block construction for approximately 13 years, from 1988 to 2003. For 2 years during this period he did work other than welding. In 2001, 12 years later, he visited a general hospital with loss of smell and was diagnosed with anosmia as a result of olfactory identification and threshold tests. Brain MRI and PNS CT revealed chronic sinusitis with mucosal thickening. He smoked approximately one pack of cigarettes per day, for 22 years.

Workplace environment measures revealed welding fumes (Max: 30.21 mg/m^3^, Min: 9.44 mg/m^3^, TLV-TWA: 5 mg/m^3^), cadmium (metal dust, Max: 0.0011 mg/m^3^, Min: N/A, TLV-TWA: 0.01 mg/m^3^), and chromium (Max: 0.0124 mg/m^3^, Min: 0.0004 mg/m^3^, TLV-TWA: 0.5 mg/m^3^). Exposure assessment was not conducted at the time of the investigation.

#### Commentary

The worker performed welding for a total of 13 years and 2 months. Based on the results of previous workplace environment evaluations, the worker was exposed to high concentrations of welding fumes (30.21 mg/m^3^) during the welding process, but the exposure levels of heavy metal dusts, such as cadmium (0.0008 mg/m^3^) and chromium (0.0075 mg/m^3^), known to be associated with olfactory disorders were low. Although exposure to welding fumes can cause olfactory disorders, Investigators noted that it was difficult to identify fumes as a leading cause of anosmia, given that chronic sinusitis was identified on the worker’s PNS CT scan. Investigators determined that the burden of occupational exposure was insufficient to cause anosmia and that the worker’s anosmia was likely related to his past medical history.

### Case 6 (F/47): Spray adhesive labeling job, volatile organic compounds

This worker performed product inspection and labeling at an automobile parts manufacturing plant for one year, from 2014 to 2015. In May 2015, a year after she attached several sheets of paper labels to product boxes using spray adhesive, her olfaction and taste decreased. After that, she underwent olfactory identification and threshold test results and was diagnosed with anosmia. There were no abnormal findings on endoscopy and PNS CT. There was no past history of head trauma, otorhinolaryngological disorders, smoking or binge drinking.

The workplace environmental evaluation revealed five organic solvents, including 2-methylpentane, but the exposure levels were as low as 0.041 ppm, or less. The estimated exposure time to organic solvents was very low because the total time during which spray adhesive was applied was very short. There was another task that involved spraying rust preventive oil with a sprayer, so that the product did not rust during packing, but it was used only for a small duration of time, during work hours, and its measured levels were approximately 10% of the permissible exposure limit.

#### Commentary

The worker performed spray adhesive labeling for approximately 11 months. She was exposed to organic solvents and rust preventive oil during work, but the concentrations of these substances were estimated to be very low, and the work time was short. Investigators determined that the burden of occupational exposure was insufficient to consider her anosmia as work related, and there was no accidental exposure to cause the acute olfactory disorder.

### Case 7 (F/50): Automobile parts epoxy coating, organic solvent

The worker performed epoxy coating in an automobile parts plant for 2 years, from 2011 to 2013. Beginning in June 2013, her olfaction began to diminish. After completing olfactory identification and threshold testing she was diagnosed with anosmia. Endoscopy was unremarkable and there were no abnormal findings on PNS CT. There was no past history of otorhinolaryngological disease and no other illnesses. There was no history of smoking or binge drinking.

Investigators determined that she could not be exposed to organic solvents when applying epoxy resin, during assembly of gas sensors. Exposure to polyoxypropylene diamine, the main component of the hardener, and methanol was likely very low, or non-existent. The soldering operation was conducted in another workplace, and the exposure levels were very low, although it was possible to be indirectly exposed to lead, tin, and silver.

#### Commentary

The worker performed epoxy resin coating for approximately 2 years. She had presumed exposure to polyoxypropylene diamine, methanol, and metal fumes while working, but the concentrations of these chemicals were estimated to be very low and the exposure period prior to symptom onset, was between 1 and 2 years. She had no history of accidents that could have caused acute anosmia. Investigators determined that the burden of occupational exposure was insufficient to cause anosmia.

### Case 8 (F/57): Clean room cleaning, organic compound (cyclohexanone)

The worker performed clean room cleaning of a mobile phone parts manufacturing area for one year, from 2013 to 2014. Beginning in September 2013, approximately 7 months after starting of work, her olfaction began to diminish. After olfactory identification and threshold testing, she was diagnosed with anosmia. There was no past history of otorhinolaryngological, or other, disease. There was no history of smoking or binge drinking.

The worker handled cyclohexanone to remove chemicals from the floor while cleaning the room. From past work environment measurement results, it appeared that cyclohexanone was universally used. According to the worker’s statement, she used 300–500 mL cyclohexanone, for approximately 1 h per day. Exposure assessment was not possible at the time of the survey, and a level of 15 ppm was assumed as per the stimulation threshold. She had detected stimulus odors but did not exhibit ophthalmologic or neurological symptoms of cyclohexanone exposure.

#### Commentary

The worker cleaned the room for about a year, during which time she was exposed to cyclohexanone. This exposure was not significantly higher than the estimated typical, daily life exposure. The exposure period until the onset of the symptoms was short, and there were no accidents that could have caused acute anosmia. The investigators determined that the burden of occupational exposure was insufficient to cause anosmia.

## Discussion

Olfactory disorder refers to a loss in the ability to smell or a change in the way odors are perceived. Hyposmia refers to a reduction in the sense of smell. Anosmia refers to a difficulty or inability to perceive odor. Although anosmia can be temporary, traumatic anosmia can induce permanent disability of olfactory function. Olfactory function testing consists of an olfactory identification test and an olfactory threshold test. The olfactory identification test is a subjective test examining if one can distinguish odors, and the olfactory threshold test determines the threshold of nerve response to olfactory chemical stimuli.

The University of Pennsylvania Smell Identification Test (UPSIT) and Cross-Cultural Smell Identification Test (CCSIT) examine the identification functions of olfaction. The Butanol Threshold Test (BTT) examines nerve response thresholds for olfactory stimuli, and The Korean Version of the Sniffin’ Sticks Test II (KVSS II) simultaneously evaluates both the olfactory identification and threshold. BTT and KVSS II are objective tests for olfactory function that can be used clinically. The olfactory results from the KVSS II and BTT tests have strong positive correlations [5]. However, it is difficult to accurately evaluate olfactory capacity solely from olfactory function tests. The KVSS II, commonly used in Korea, was modified into a Korean version after its development in Europe and is relatively well known in Korea [6]. The KVSS II test consists of three major tests: the olfactory threshold test, the smell discrimination test, and smell identification test. The smell identification test of the KVSS II features high test–retest reliability; however, the other two tests (the threshold test and the discrimination test) do not [7]. The mean KVSS II score was 30.37±4.75 for normosmia, 23.82±6.81 for hyposmia, and 10.69±3.37 for anosmia. In all the present cases, anosmia was diagnosed after repeated tests.

Olfactory disorder can be sub-classified according to its cause: conductive olfactory nerve dysfunction in which chemical molecule delivery to olfactory cells is impaired and sensorineural olfactory disorder in which olfactory cells or the nerve itself is impaired [8].

Anosmia can occur when the structures inside nasal cavities deteriorate or become damaged due to chronic sinonasal inflammation or when the axons of olfactory receptor cells become damaged due to head injury. In addition, olfactory disorder can result from mental disorders, poor nutritional status, irradiation, and specific drugs that inhibit the regeneration of receptor cells or affect the secretion around the olfactory mucosa and therefore induce mucosal changes. Furthermore, chronic olfactory disorder can be age-related. Aging leads to loss of olfactory receptor cells and replacement of olfactory epithelium with respiratory epithelium [9]. Toxic substances in occupational settings, which are known to trigger olfactory disorders by affecting the olfactory mucosa, include heavy metals, carbon tetrachloride, carbon monoxide, sulfurous acid gas, formaldehyde, organic solvents, and benzene [10]. Cases of olfactory disorder, caused by metal and chemical substances, have also been reported [11, 12]. CT scan can be used to detect abnormalities in the sinuses, cranium and its base, and nasal cavities. When patients have a history of head injury, neurological manifestations, or Kallmann syndrome or when sinus infections or neoplasms are suspected, MRI can be used to detect disturbances in the brain, soft tissues, and sinuses. However, clinical imaging assessments may be of limited use for patients with idiopathic anosmia of an undetermined cause. According to a retrospective cohort study that examined 839 patients with anosmia, MRI was performed in 55% of cases to determine the cause, but the abnormalities that could explain the patients’ anosmia were determined in only 0.8% of patients [13]. In addition to MRI, blood tests can be used to determine other medical conditions that could induce anosmia or detect exposures to heavy metals. In the previous cases where sinusitis was detected from CT and a history of trauma existed, anosmia was likely caused by underlying diseases. In Cases 1, 2, and 3, organic solvents contributed to the anosmia because the burden of occupational exposure was sufficient. In Cases 4 and 5, although there was a suspicion of exposure to organic solvent and welding fumes, respectively, they were regarded as low work-relatedness cases. The estimated amount of exposure was very low in both cases. The burden of occupational exposure was regarded as the most important reason for low work-relatedness, although chronic sinusitis (Case 4 and 5) is the major cause of anosmia and chronic sinusitis commonly occurs in the presence of frontal lobe encephalomalacia (Case 4) [14]. When determining the occupational relatedness, underlying disease were not considered more important than occupational burden although, anosmia was likely caused by these underlying diseases.

Although smoking can cause olfactory disorders, it was not considered as a decisive factor when there was sufficient burden of occupational exposure. Both Case 1 (5 pack-years) and 2 (15 pack-years) had a history of smoking. Occupational-relatedness was noted because the burden of occupational exposure was sufficient to cause anosmia. In all cases, occupational burden is considered to be more important than underlying disease or past history.

In the U.S., studies on the prevalence of olfactory disturbances are less common than the studies on the prevalence of dysopsia or dysecoia. According to a study by Claire Murphy et al. (2002), the San Diego Odor Identification Test, administered to 2491 adults between the age of 53 and 97 years, showed that 24.5% of the subjects had olfactory disorder. In the elderly aged 80–97 years, 62.5% had olfactory disorder, suggesting that aging is associated with olfactory disorder. Other factors such as smoking, stroke, epilepsy, and upper respiratory tract infections can also relate to anosmia. However, the prevalence of self-reported olfactory disorder was only 9.5%, which was far lower than the prevalence indicated by objective measures [15].

There has been only one study conducted by Lee et al. on the prevalence of olfactory disorder or its risk factors in Korea. They analyzed the prevalence and causes of olfactory disorder based on the 2009 Korea National Health and Nutrition Examination Survey. Their results showed that 4.5% of the 7300 study subjects complained of subjective olfactory disorders. Risk factor analysis showed that chronic sinusitis, history of hepatitis B, air pollutants, rhinitis, and low income were risk factors that were highly associated with anosmia. However, their study has limitations since the presence or absence of olfactory disorder was based on subjective responses to the survey and not on objectively measured data [16].

In the present study, we analyzed cases of anosmia, referred to the Korea Safety and Health Agency for the analysis of occupational causality, and investigated the characteristics of the cases that were potentially work-related. The average age of the workers subjected to occupational exposure was 44.3 years, and the average duration of exposure was 129 months (10.7 years). The average age of the workers who had low occupational-relatedness was 51.3 years, and the average duration of exposure was 19.3 months (1.6 years). The average age was lower, and the duration of exposure was longer in the workers with higher occupational exposures.

We were unable to quantify and compare the amount of direct exposure in all cases. However, in low-occupational exposure cases, they spent much less time in a hazardous work environment during their routine job and the duration of exposure to harmful substances was also low, leading to low-total accumulated exposure. Even when substances that can induce olfactory disorder were used, the contributions of occupational exposure were low. Specifically, the accumulated exposure, depending on the duration and concentration, was too low to cause anosmia. In addition to occupational causes, there are underlying diseases that could trigger olfactory disorders, such as brain disorders or sinus infections.

In the cases with high occupational exposure, the workers worked long hours, performing tasks that could induce olfactory disorders; they were reported to have a relatively longer exposure duration. They did not have other triggering factors such as an underlying disease. The cases that were work-related were mainly cases of anosmia that occurred after long-term exposure to organic solvents. Although there was a study in Korea on long-term exposure to organic solvents and occupational olfactory damage, there are no studies examining specific cases of occupational related anosmia. There are no known cases where anosmia occurred due to short-term occupational exposure in Korea, and there is no case that proves an occupational cause-and-effect relationship with short-term exposure to organic solvents. However, a number of anosmia cases, caused by short-term exposure, exist outside Korea. These include the case of a 57-year-old male worker diagnosed with occupational olfactory loss which lasted for 1 year after the onset of bloody nasal secretions and nasal itching that persisted for several days following 3 weeks of exposure to high-concentration barbituric acid [17]. There is an additional case of a 31-year-old male worker who developed anosmia after approximately 4 weeks of using water-proofing chemicals [18]. In our case study, there were three cases of anosmia that occurred after 1–2 years of occupational exposure to organic solvents, which is a relatively short time period. The work-relatedness was determined to be low in all of these cases. The evaluation results showed that there were no underlying diseases, and although there was a temporal order between the occupational exposure and the onset of anosmia, the measured amount of exposure was too low to damage the olfactory nerve in such a short time period, and the frequency of exposure to the substance was very low (Case 6). At times, the exposure concentration was not separately measured, and the frequency of exposure was low when the work process was analyzed (Cases 7, 8). Consequently, these cases were not recognized as occupationally related disease. In all three cases, the work-relatedness was determined to be low due to the lack of exposure to a strong stimulus that could permanently damage the olfactory nerve in a short time period.

The limitations of this study are as follows. First, this study included only cases that required investigation because the relationship between work and symptom onset was unknown. The initial symptom of olfactory disorder could be subtle and not immediately apparent, so it is difficult to distinguish whether the symptom onset is before or after the occupational exposure. Obvious olfactory damage caused by unforeseen work accidents are not investigated due to the clear relationship between work duties and olfactory loss. These cases were not included in this study and we cannot be certain of the number of such cases. We only included workers with chronic exposure. Second, because it is difficult to accurately determine the prevalence of anosmia in the general population, the criteria for determining the degree of work-relatedness were limited to duration of exposure, amount of exposure, exposure concentration, and underlying disease.

## Conclusions

This was a study on occupationally linked anosmia. The study results confirmed work-relatedness when the subject was young, and the duration of exposure was long without any other cause of anosmia. However, our study only examined work-relatedness and did not establish clear scientific evidence. Regarding compensation for occupational diseases, work-relatedness can be recognized as a relative concept. Hence, sequential and systematic studies on occupationally linked anosmia are warranted in the future.

## Abbreviations

KCOMWEL: Korea Workers Compensation and Welfare Service
KOSHA: Korea Occupational Safety and Health Agency
OSHRI: Occupational Safety and Health Research Institute
TLV-TWA: Threshold limit value/Time-weighted average
